# Chromosome-scale genome assembly of *Artabotrys hexapetalus* provides insights into its evolution and terpenoid biosynthesis

**DOI:** 10.1186/s12870-025-07645-w

**Published:** 2026-08-12

**Authors:** Zhen-Jia Cui, Dan Ye, Yun-Bao Liu, Ping Zhu, Shi-Shan Yu

**Affiliations:** 1 State Key Laboratory of Bioactive Substance and Function of Natural Medicines, Institute of Materia Medica, Chinese Academy of Medical Sciences & Peking Union Medical College, Beijing, 100050 People’s Republic of China; 2 NHC Key Laboratory of Biosynthesis of Natural Products, Institute of Materia Medica, Chinese Academy of Medical Sciences & Peking Union Medical College, Beijing, 100050 People’s Republic of China

**Keywords:** Artabotrys hexapetalus, Genome, Terpene biosynthesis

## Abstract

*Artabotrys hexapetalus* is used as traditional Chinese medicine for the treatment of malaria. Its antimalarial activity has been shown to be the result of the sesquiterpene designated as yingzhaosu A (YZSA), which mainly occurs in the roots of A. hexapetalus. Despite its medicinal significance, the genetic basis and molecular mechanisms underlying the biosynthesis of its active terpenoids remain unclear. In this study, a high-quality chromosome-scale genome of *A. hexapetalus* was first reported. In total, 915.15 Mb of genome sequences were assembled, of which 911.58 Mb was sorted into 8 chromosomes. Phylogenetic analysis indicated that the divergence between the genera Artabotrys and Annona happened at approximately 45.77 million years ago (Mya) and the Annonaceae family diverged from other Magnolia species about 103.58 Mya. An ancient whole genome duplication (WGD) event was discovered in the *A. hexapetalus* genome, earlier than the divergence between Annonaceae and Magnoliaceae families. It correlates with a significant expansion of gene families related to secondary metabolism, especially the terpene synthase (TPS) and cytochrome P450 (P450) families. The analysis of TPS, P450, and 2-oxoglutarate/Fe(II)-dependent dioxygenase (2-ODD) gene families provides a foundation for future functional studies. Additionally, three putative terpene biosynthetic gene clusters were identified within the genome. Our work provides a foundation for elucidating the complete biosynthetic pathway of terpenoids in *A. hexapetalus* in the future.

**Supplementary Information:**

The online version contains supplementary material available at 10.1186/s12870-025-07645-w.

## Introduction

*Artabotrys hexapetalus*, a woody climber of the Annonaceae family, has been used as traditional Chinese medicine for the treatment of malaria. Previous studies have identified multiple secondary metabolites, including sesquiterpenoids [1–4], alkaloids [5–7], flavonoids [8–10] and butyrolactones [11] from *A. hexapetalus* and other *Artabotrys* species, and documented various pharmacological activity, such as antimalarial [12, 13], antiviral [4, 14], antimicrobial [15–17], cytotoxic [4–6, 8] and antioxidant [17, 18] effects. Among them the most notable antimalarial activity is attributed to the presence of yingzhaosu A (YZSA), an endoperoxide-containing bisabolane-type sesquiterpene. YZSA was first isolated in 1979 in the roots of the *A. hexapetalus* [1], exhibiting potent inhibitory effects against *Plasmodium falciparum* [12, 13]. Besides, a recent study developed probes from the unique reaction between heme and YZSA, and subsequently applied the probe to identify ring-stage inhibitors of *P. falciparum*, thus broadening its application [19]. However, YZSA exists in trace amounts in *A. hexapetalus,* which is also considered as a rare resource, making it difficult to obtain large quantities through traditional extraction methods. Although significant efforts have been devoted to improving chemical synthesis, current routes are limited to yields of 7.3% due to the structural and stereochemical complexity of YZSA [12, 20], thus elucidating its biosynthetic pathway is significant for overcoming source limitations and promoting its applications.

With recent improvements in the quality and cost-effectiveness of genome sequencing, it has become an indispensable tool for studying plant natural products in unexplored non-model species [21, 22]. As the second largest family in Magnoliales and one of the most species-rich pantropical plant families, the Annonaceae has five reported draft genomes so far [23–27]. The soursop (*Annona muricata*) genome, representing the first genome assembled for the family, not only improves the understanding of Annonaceae genetic diversity and phylogenetic position, but also provides novel data on disease resistance and plant defense for the breeding of other tropical trees [23]. Besides, *Annona cherimola* genome research aims to facilitate breeding programs to increase its resilience under different climate change scenarios, with an ultimate goal of increasing food security in regions with subtropical climate [24]. Research on the perfume tree *Cananga odorata* is focused on the molecular mechanisms involved in the biosynthesis of the floral volatile organic compounds (VOCs), the source of its aroma [25]. *Annona glabra* genome was sequenced in a study to explore the evolution and adaptability of coastal mangrove populations based on a full set of mangrove genomes [26]. Besides, a recent study presents the complete genome sequence of *Annona squamosa* [27]. High-quality genomic resources for *A. hexapetalus* would facilitate phylogenetic studies on Annonaceae and expedite the exploration of biosynthetic pathways of related active natural products.

In plants, the biosynthesis of terpene is carried out by terpene synthases (TPSs), which are key enzymes in terpenoid metabolism, generating the structural diversity of the core terpene skeleton. Sesquiterpene is typically cyclized from the common precursor C15 farnesyl diphosphate (FPP) via the action of sesquiterpene synthases. Several studies have reported the biosynthetic enzyme of bisabolene [28–31], the precursor of YZSA. However, the tailoring enzymes such as P450 and 2-ODD involved in further biosynthesis of the unique endoperoxide in YZSA remain enigmatic. P450s form the largest enzyme family in plants, serving as ubiquitous tailoring enzymes that are capable of catalyzing diverse oxidation reactions, including hydroxylation [32, 33], epoxidation [34] and cyclization [35, 36]. P450s can be divided into 11 clans, among them CYP71 family has been reported mainly involved in the biosynthesis of plant sesquiterpenes [37]. 2ODDs are soluble, nonheme iron-containing enzymes, which could be classified into three major classes: DOXAs, DOXBs and DOXCs, and among them DOXC enzymes largely participate in plant secondary metabolism [38]. In plants, 2-ODDs catalyze a wider range of reactions including ring formations, rearrangements, desaturations, and halogenations [39]. Recently, a few enzymes were reported to form endoperoxides, including COX from animals [40], FtmOx1 from *Aspergillus fumigatus* [41] and NvfI from *Aspergillus novofumigatus* [42], with the latter two being 2-ODDs. Additionally, the iodide peroxidase in the extract of *Chenopodium ambrosioides* fruit catalyzes the transformation from *α*-terpinene to ascaridole, a process that involves the formation of a peroxide bond [43]. However, the specific enzyme responsible for this peroxide bond formation remains unclear.

In this study, a high-quality chromosome-scale genome of *A. hexapetalus* was reported for the first time. In total, 915.15 Mb of genome sequences were assembled, of which 911.58 Mb were sorted into 8 chromosomes. Phylogenetic analysis suggested the divergence between the genera *Artabotrys* and *Annona* happened at approximately 45.77 million years ago (Mya) and the Annonaceae diverged from other Magnolia species about 103.58 Mya. An ancient WGD event was discovered in the *A. hexapetalus* genome, earlier than the divergence between Annonaceae and Magnoliaceae families. Significant expansion of gene families was found involved in secondary metabolites, especially the TPS and P450 families. We analyzed the TPS, P450 and 2-ODD families associated with sesquiterpene biosynthesis in *A. hexapetalus* genome, providing baseline data for future functional characterization. Besides, we also discovered three putative terpene biosynthetic gene clusters in the genome of *A. hexapetalus*.

## Results

### *Artabotrys hexapetalus* genome assembly and annotation

**Fig. 1:**
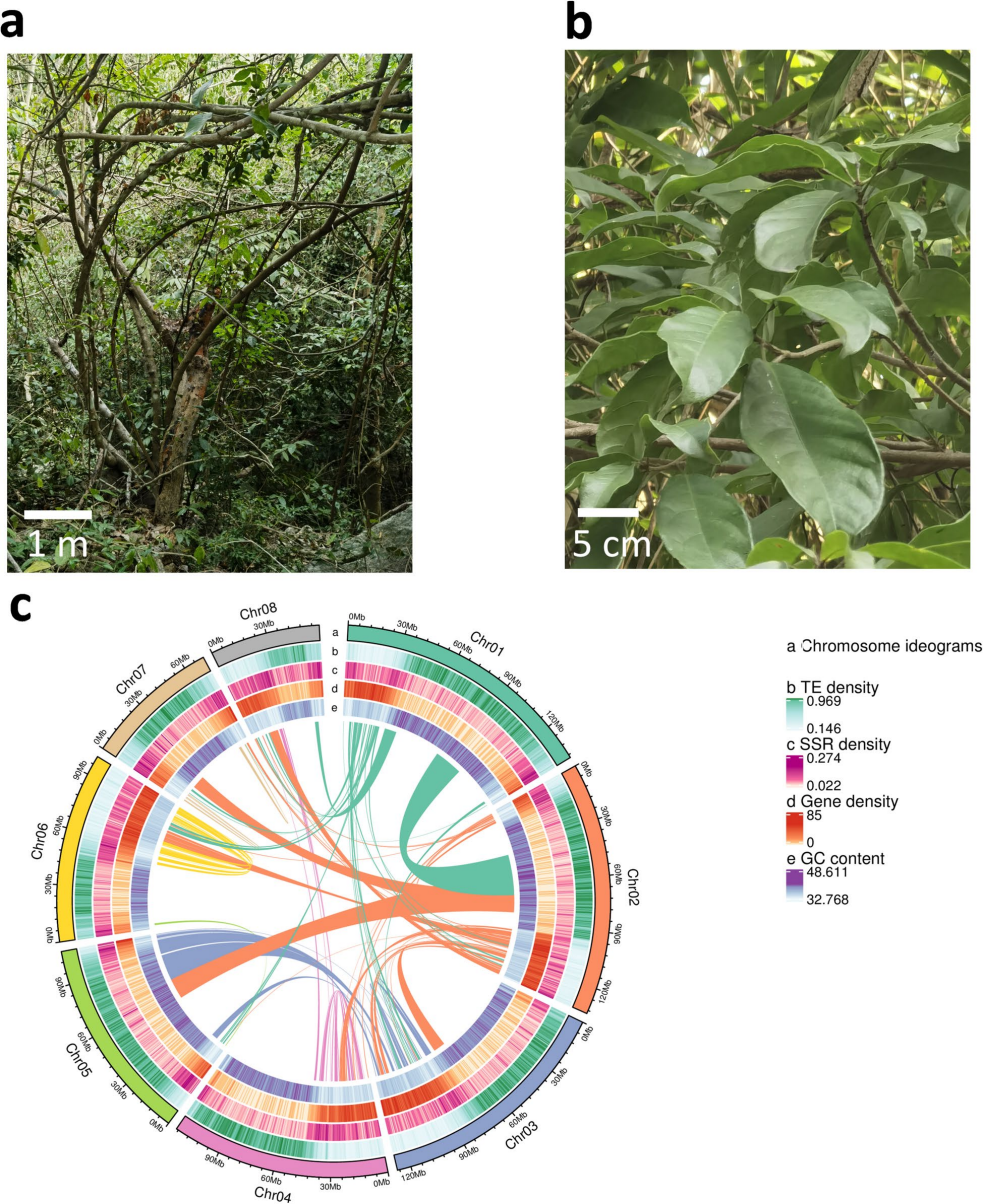
*A. hexapetalus* description and genomic landscape. **a** Whole plant; **b** leaves; **c** Overview of *A. hexapetalus* genome assembly features. The outer track represents chromosomes. Tracks from b to e represent the distribution of TE density, SSR density, gene density and GC content, respectively. The inner circle represents syntenic gene pairs within *A*. *hexapetalus* genome

Tender leaves of *Artabotrys hexapetalus* planted in Hainan province were selected for genome assembly (Fig. 1a and b). A high-quality assembled genome of *A*. *hexapetalus* was generated by combining Illumina short-read sequencing, PacBio SMRT (single molecule real time) sequencing and Hi-C (high-throughput chromosome conformation capture) technology. Based on *k-mer* analysis, the genome size of *A*. *hexapetalus* was estimated to be 892.37 Mb with a relatively high level of repeats (59.05%) and low level of heterozygosity (0.24%) (Figure S1; Table S1). Therefore, the genome of *A*. *hexapetalus* is a simple genome, facilitating the subsequent construction of a detailed genomic map. A total of 915.15Mb sequences of *A*. *hexapetalus* genome were assembled using 72.62Gb PacBio long reads (79.35 × coverage) and aligned with 102.26 Gb Illumina whole-genome sequencing reads (114 × coverage) to assess the completeness (Tables S2 and S3). To refine and obtain a chromosome-scale assembly, the Hi-C-Pro program was further used to produce chromosomal interaction maps with Hi-C reads mapped to the preliminary assembly, and 911.58 Mb of the assembled genome was anchored into 8 chromosomes (Figs. 1c and S2; Table S4 and S5), which covered 99.61% of the genome. Furthermore, BUSCO (Benchmarking Universal Single-Copy Orthologs) analysis showed that 97.40% of the gene sets were detected, illustrating a high completeness of gene prediction (Table S6).

Based on genomic information, 23,859 protein-coding genes with an average gene length of 5626.98 bp and an average of approximately 6 exons per gene were further annotated by integrating transcriptome data, homologous alignments and ab initio gene models (Table 1; Tables S7 and S8). Most genes are supported by at least two prediction methods, indicating a high quality of prediction (Figure S3). Functional annotation was performed using NR, eggnog, GO, KEGG, TrEMBL, KOG, SWISSPROT and Pfam databases, and 97.06% (23158) of the predicted protein-coding genes had been annotated (Table S9).

**Table 1:** Genomic features of *A*. *hexapetalus*

Genomic features	Data value
Contig Number	984
Contig Length (bp)	915,145,870
Contig N50 (bp)	3,810,937
GC(%)	38.02
Gap Number	463
Number of transcripts	23,859
Total size of TEs (bp)	628,663,483
BUSCO (%)	97.40%

The noncoding RNAs in the *A. hexapetalus* genome were also identified, including 551 tRNAs, 57 microRNAs, 236 rRNAs, 59 snRNAs and 81 snoRNAs (Table S10). Moreover, the repeated sequences were also identified including tandem repeats accounting for 7.29% of the genome and transposable elements accounting for 68.69% of the genome (Table S11 and S12). Among transposable elements, LTRs (long terminal repeats) were the most abundant repetitive elements, accounting for 60.50% of the total genome sequences, and most of the LTRs were Gypsy (44.30%) and Copia (5.42%) elements (Table S11). The ratio of Gypsy to Copia (8.18) was much higher in the *A. hexapetalus* genome than that in close relatives *Annona cherimola* (1.23) [24], suggesting that the Gypsy transposon amplification was a key driver for the evolution of the *A. hexapetalus* genome. Furthermore, the intact LTR insertion events in the *A. hexapetalus* genome were observed as a continuous process, with most of the insertions having occurred in the last 2 million years (Figure S4).

### Evolution of the *A. hexapetalus* genome

**Fig. 2:**
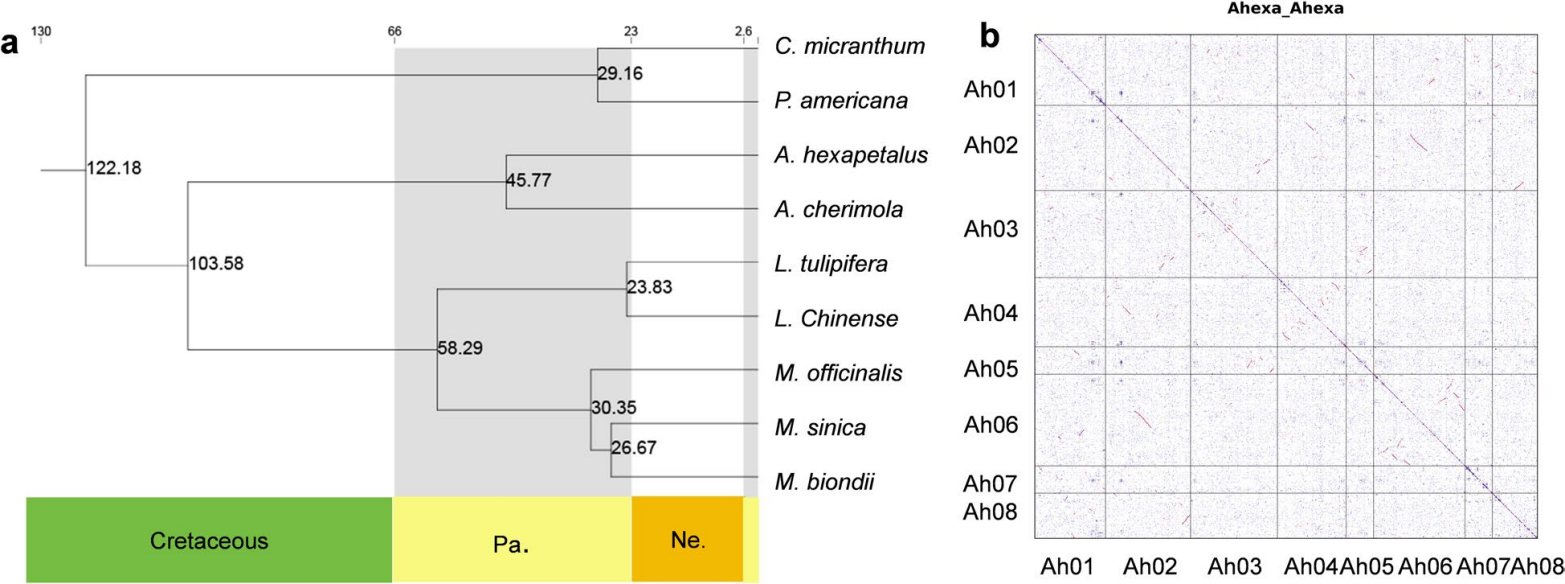
The evolution of *A. hexapetalus* genome. **a** Phylogenetic relationships and estimated divergence time among nine plant species. The phylogenetic tree was constructed using 1111 single-copy genes shared by all species used in current study, and the estimated divergence times supported by 95% of the HPD (highest posterior density) are displayed by the black number at each branch node. At the base of the tree is the geological time, and at the top of the tree is the absolute age, measured in millions of years, with the shade defining each geological period. Pa., Paleogene, Ne., Neogene. **b** Syntenic dot plots of the paralogs in *A*. *hexapetalus* genome

The evolutionary history of *A*. *hexapetalus* was investigated based on the assembled and annotated genome. A set of 1111 single-copy orthologs from 9 angiosperm species (Table 2 and S13) was identified using OrthoFinder. According to sequence alignment of these single-copy orthologs, a phylogenetic tree was constructed, *C. micranthum* and *P. americana* were assigned as outgroups (Fig. 2). The accuracy of the phylogenetic tree was supported by bootstrap values with a minimum support rate of 100 (Figure S5). This phylogenetic tree showed that *A*. *hexapetalus* was clustered with the other Annonaceae species *A. cherimola* as expected, while this group diverged from their most recent common ancestor approximately 45.77 million years ago (Mya). And Annonaceae family diverged from other Magnolia species about 103.58 Mya.

**Table 2:** Angiosperm species in this study

Species	Abbreviation for species
*Phytolacca americana L*	*P. americana*
*Magnolia officinalis*	*M. officinalis*
*Cinnamomum micranthum (Hayata) Hayata*	*C. micranthum*
*Artabotrys hexapetalus*	*A. hexapetalus*
*Magnolia sinica*	*M. sinica*
*Magnolia biondii*	*M. biondii*
*Liriodendron tulipifera L*	*L. tulipifera*
*Liriodendron chinense (Hemsl.) Sarg*	*L. chinense*
*Annona cherimolia Mill*	*A. cherimola*

**Fig.3:**
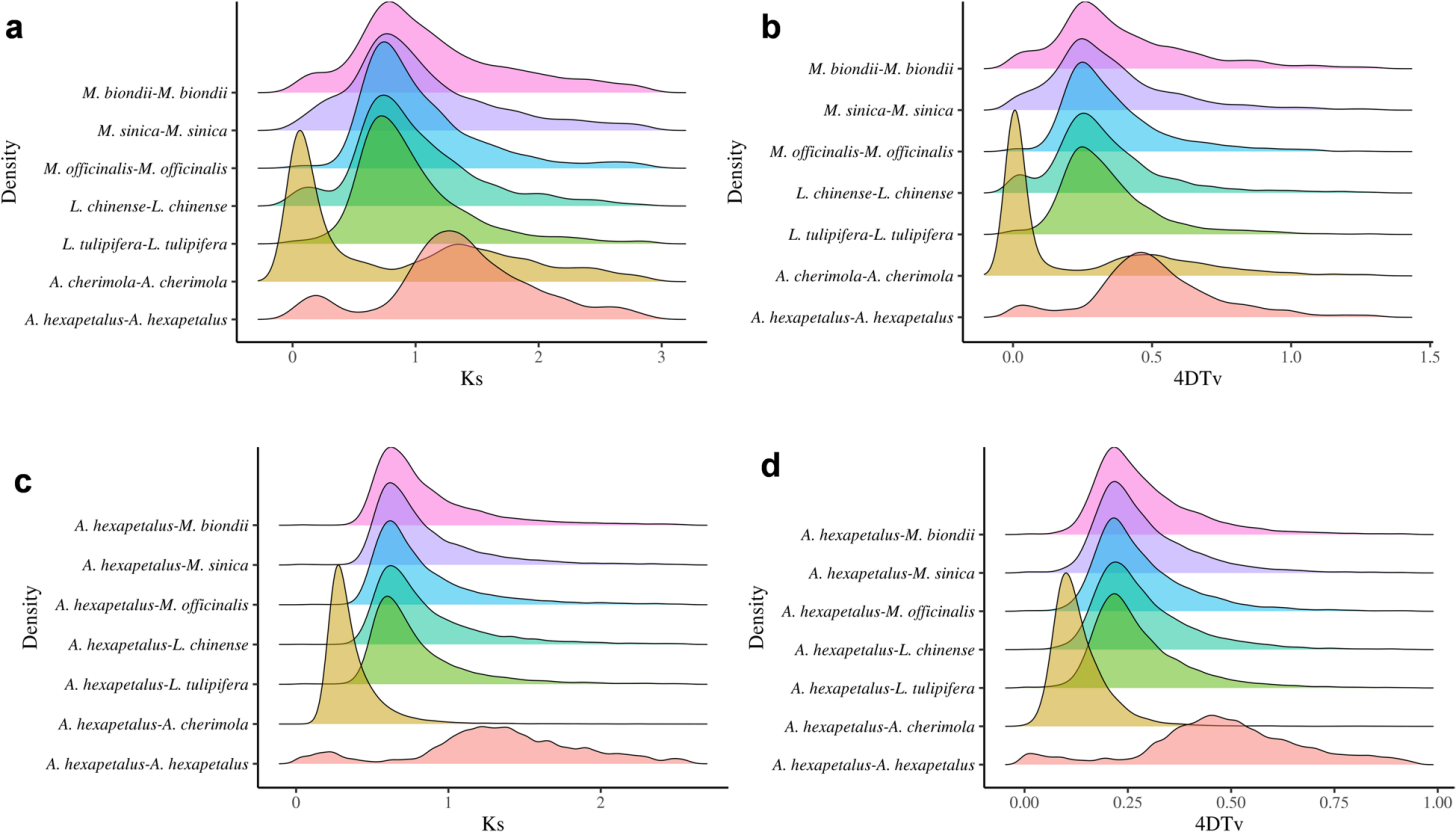
Whole-genome duplication analysis of *A*. *hexapetalus*. A larger value on the x-axis indicates a greater distance from the present time, implying that it is more ancient. **a** Ks values Distribution map of *A. hexapetalus* and other indicated species. **b** 4DTv Distribution map of *A. hexapetalus* and other indicated species. **c** Ks values Distribution map of one vs one ortholog comparison between *A. hexapetalus* and other indicated species. **d** 4DTv values Distribution map of one vs one ortholog comparison between *A. hexapetalus* and other indicated species

Given that WGD event has been recognized as an important evolutionary force in plants contributing to the expansion of plant genome size [44], it was investigated whether *A. hexapetalus* had experienced any WGD events. Firstly, the synteny within the *A*. *hexapetalus* genome was analyzed using MCScanX [45], and 119 syntenic blocks were found throughout the whole genome, containing 3690 collinear genes (Figs. 1c and 2b; Table S14). The synonymous substitutions per synonymous site (Ks) distribution of paralogous gene pairs was further analyzed throughout the entire *A*. *hexapetalus* genome. The result showed a signature peak at approximately 1.29 (Fig. 3a), indicating that an ancient WGD event had occurred, essentially concurrent with that in *Annona cherimola*, which is also in the Annonaceae. Additionally, a minor peak at around 0.22 was detected, which likely represent background small scale gene duplication rather than recent WGD. Moreover, the 4DTv (four-fold degenerate synonymous third-codon transversion rate) value was also calculated to further analyze WGD events and a major peak was detected at 0.47 and a minor peak at approximately 0.07 (Fig. 3b), consistent with the conclusions drawn from the Ks analysis. The results of one vs one ortholog comparison with the other species suggested that the WGD event in *A*. *hexapetalus* is earlier than the divergence between the Annonaceae and Magnoliaceae families at around 0.63Ks units, 0.22 4DTv units (Fig. 3c and d).

### Gene family evolution elevated secondary metabolism in *A. hexapetalus*

**Fig. 4:**
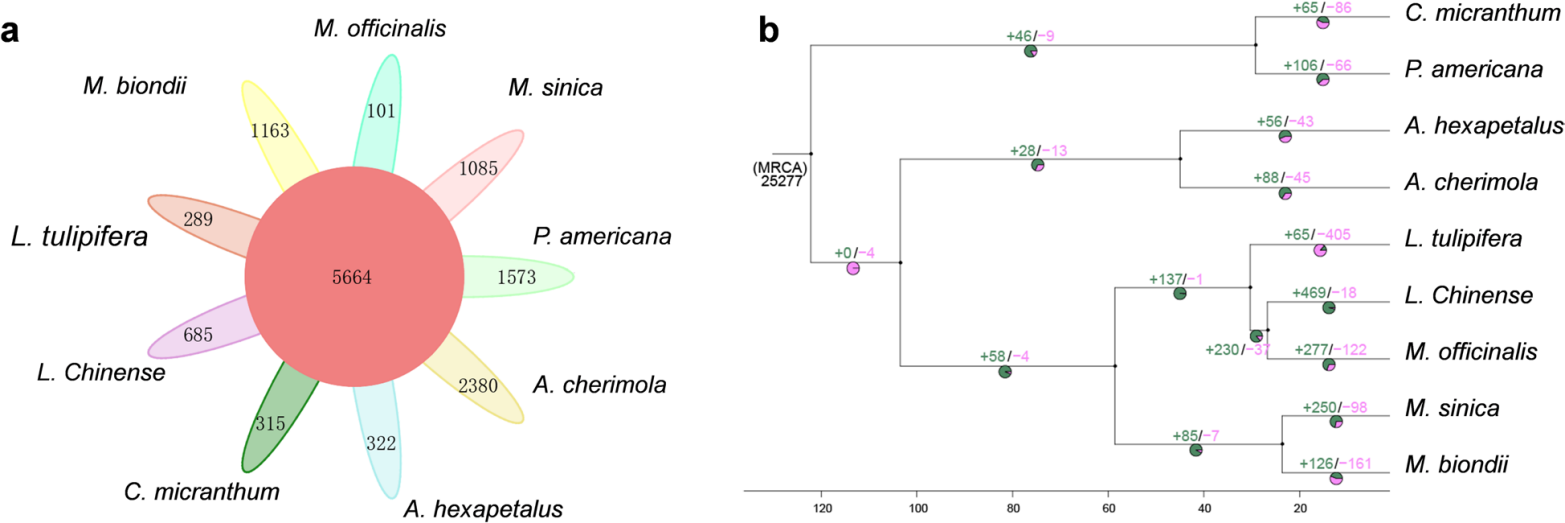
Gene family evolution in *A*. *hexapetalus.* **a** Gene family clustering flower plot. **b** Contraction and expansion of gene families for each branch of the evolutionary tree. “ + ” represents the number of gene families that expanded on that node or branch, and “-” represents the number of gene families that contracted on that node or branch

**Fig. 5:**
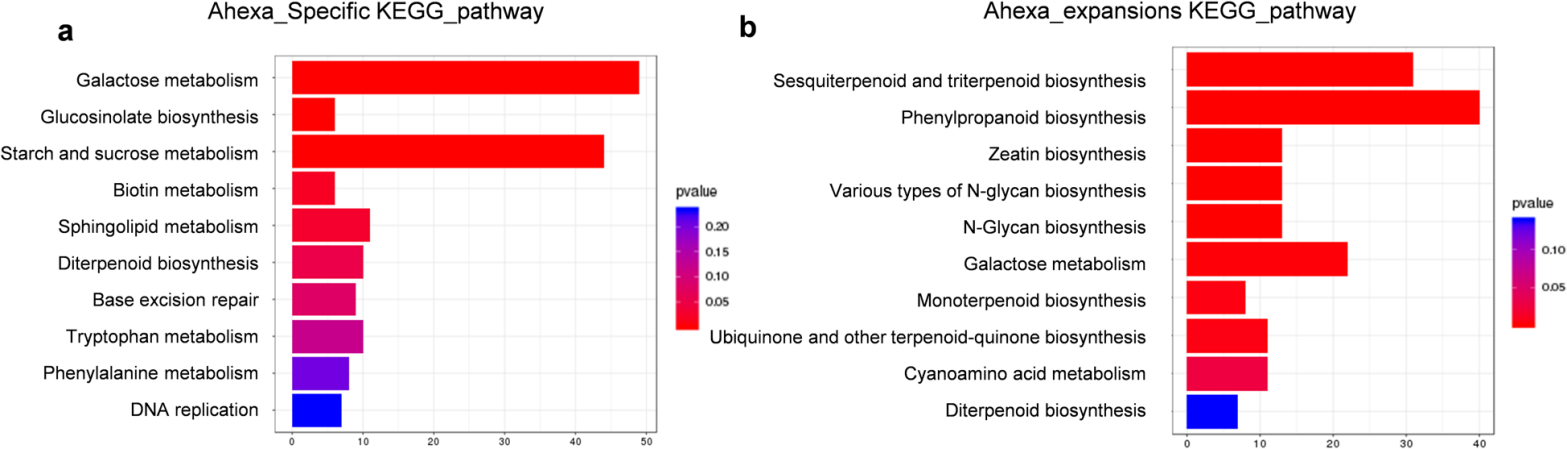
The KEGG functional enrichment of *A*. *hexapetalus* genes. **a** The KEGG functional enrichment of *A*. *hexapetalus* specific genes. **b** The KEGG functional enrichment of *A*. *hexapetalus* expansion genes

To understand the context of metabolic networks during *A*. *hexapetalus* evolution, the evolution of gene families was investigated by comparing orthologous genes between *A*. *hexapetalus* and 8 other sequenced angiosperm species. Grounded in orthologous clustering analysis, a total of 33197 orthologous gene families were identified. Among them, 5664 gene families were common to all 9 species, and 322 gene families comprising 2272 genes were unique to *A*. *hexapetalus* (Fig. 4a; Table S15). Subsequently, functional enrichment analysis of *A*. *hexapetalus* unique genes using GO terms revealed that many unique genes were grouped into metabolic process and catalytic activity catalogues (Figure S6; Table S14), indicating possible species-specific biosynthesis processes, leading to unique secondary metabolites. KEGG functional enrichment analysis results indicated that the specific genes of *A*. *hexapetalus* are mainly involved in two pathways: galactose metabolism, as well as starch and sucrose metabolism (Figs. 5a and S7).

Based on the results of gene family cluster analysis, the expansion and contraction of gene families in *A*. *hexapetalus* were explored using CAFE [46], which revealed that 56 gene families have expanded after divergence from *A. cherimola*, meanwhile 43 gene families have contracted (Fig. 4b). Functional annotation demonstrated that numerous genes were enriched in TPS family and CYP76 family involved in secondary metabolite biosynthesis, supporting that terpenes are important specialized metabolites in *A*. *hexapetalus* and the expanded TPS family and CYP76 family likely contribute to terpene diversity in *A*. *hexapetalus.* (Table S17). The expanded gene families in *A. hexapetalus* were found to be enriched in enriched in several KEGG pathways associated with specific secondary metabolism, such as “sesquiterpenoid and triterpenoid biosynthesis”, “phenylpropanoid biosynthesis”, “N-glycan biosynthesis”, “monoterpenoid biosynthesis”, “ubiquinone and other terpenoid-quinone biosynthesis” and “diterpenoid biosynthesis” (Figs. 5b and S8). It is suggested that distinct species-specific biosynthesis processes may lead to unique secondary metabolites.

### Identification of TPS gene members in* A. hexapetalus* genome

**Fig. 6:**
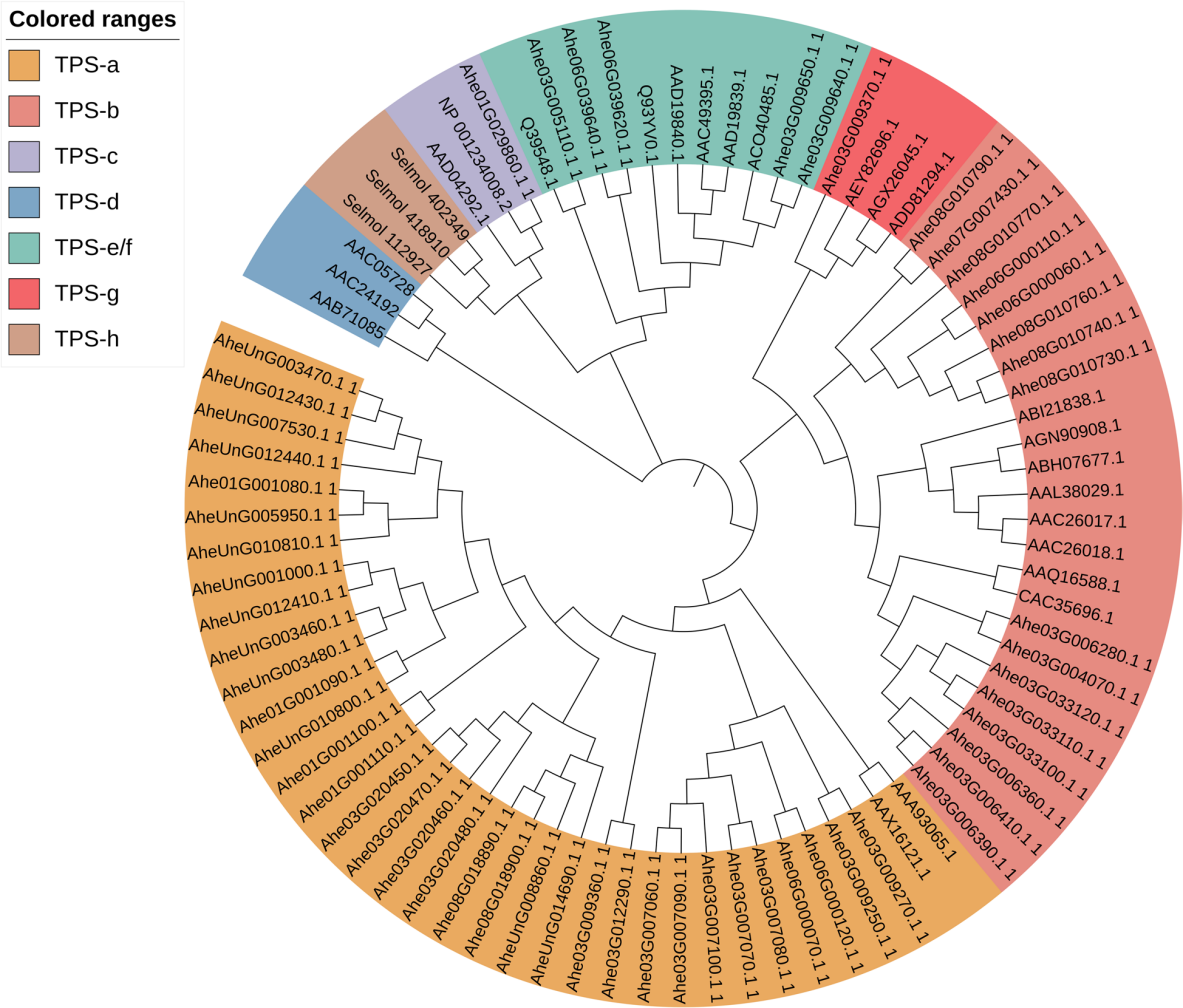
Phylogenetic analysis of *A*. *hexapetalus* terpene synthases genes based on their predicted protein sequences

Terpenes comprise the most chemically and structurally diverse family of natural products. TPS catalyzes a limited number of oligoprenyl diphosphates to terpene skeleton, in which more than half of the substrate carbon atoms undergo changes in bonding and hybridization during a single enzyme-catalyzed cyclization reaction [47]. *A*. *hexapetalus* contains various active terpenoid compounds, especially the sesquiterpene YZSA distinguished for its antimalarial activity. To explore its terpenoid biosynthesis pathway, a total of 57 TPSs containing both PF01397 and PF03936 conserved domains were identified in *A*. *hexapetalus* genome [48], of which 44 are mapped on chromosomes (Figure S9). Phylogenetic analysis based on protein sequences was performed using 57 AhTPSs and another 27 documented TPSs from different plant species (Table S18) [49–52]. In addition to the phylogenetic tree, the characteristics of structural domains and motifs (Figure S10), along with online BLAST results, were also considered to determine the family classification of each AhTPS gene. These results indicated that AhTPS genes belong to six subfamilies from TPS-a to TPS-g except TPS-d (Fig. 6 and Table S19). In *A. hexapetalus*, the TPS-a gene subfamily is the most expanded, containing 34 genes, which accounts for approximately 59.65% of the total TPS genes identified (Table S19). This pattern is consistent with observations in other plant species [52]. TPS-b gene subfamily is the second largest, including 16 TPSs (28.07%) (Table S19). The analysis also revealed that the chromosomal locations of AhTPSs were characterized by substantial gene clusters (Table S20). On chromosome 1, four TPS genes from the TPS-a subfamily occurred in a 234-kb long cluster, while another 5 TPS-b genes were located on chromosome 3 as a cluster in a stretch of 200.43 kb (Table S20). In addition, 4 TPS genes divided into TPS-a and TPS-b groups exist in a cluster on chromosome 6, with the identity between the two genes in each group as 95.63% and 98.90%, respectively (Table S20), suggesting a gene duplication event. As it has been reported that TPS-a subfamily is primarily responsible for the sesquiterpene cyclization function in angiosperms, with some members of the TPS-c and TPS-e/f subfamilies also contributing to this process [52], further insight can be gained into TPSs that may be involved in active sesquiterpene biosynthesis.

### Identification of oxygenase gene members in *A. hexapetalus* genome

**Fig. 7:**
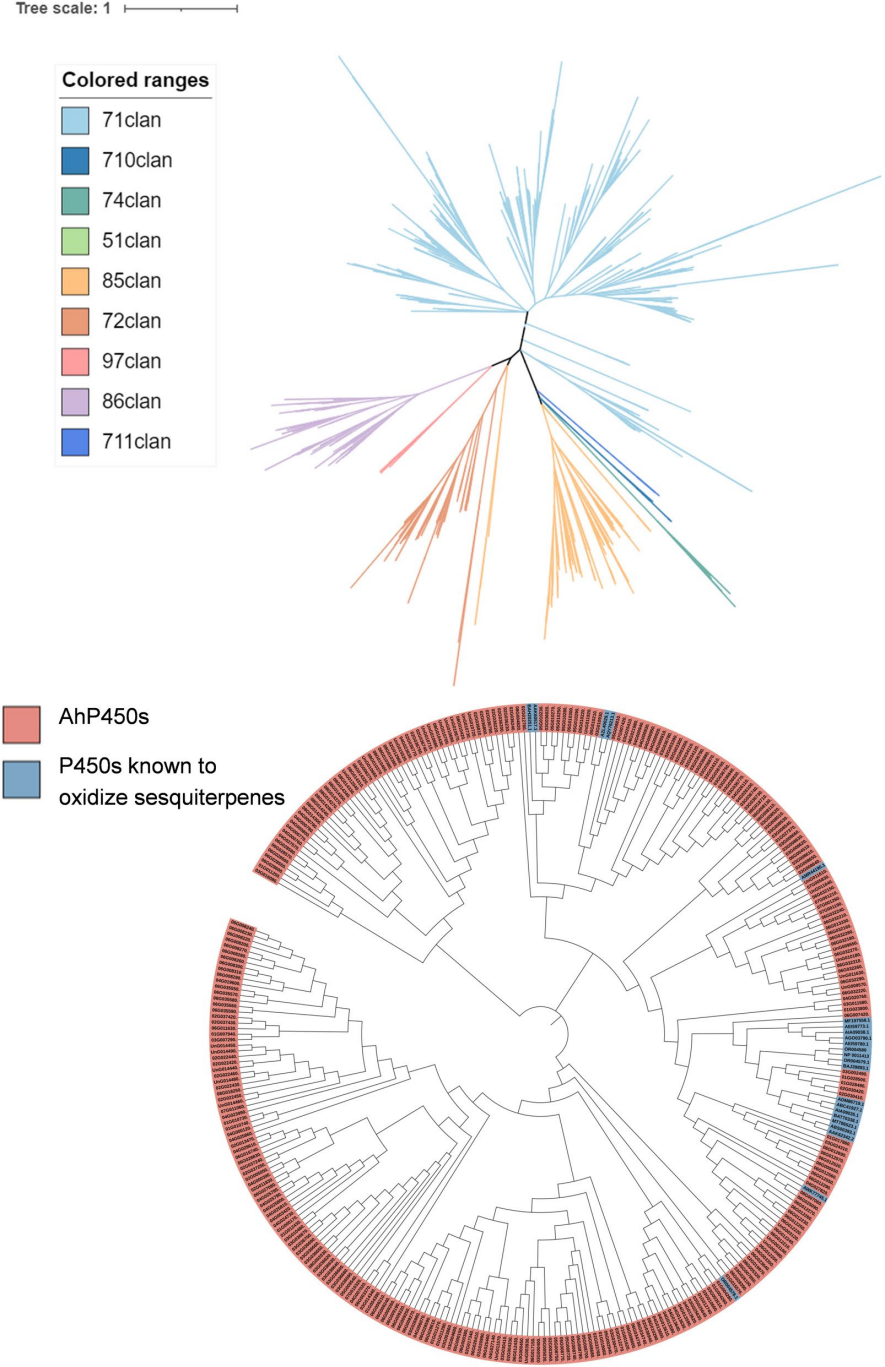
Analysis of P450 candidates. **a** The unrooted ML phylogenetic tree shows that all AhP450s can be divided into nine clans, each clan is presented by different color branches. **b** ML tree of AhP450s and P450s known to oxidize sesquiterpenes

To explore the biosynthesis of highly oxidized terpenoids abundant in A. hexapetalus, P450 and 2-ODD in its genome were analyzed, as they are the two most abundant types of oxygenases in nature. A total of 310 P450 genes containing PF00067, a typical CYP motif in plants [53], were identified in the *A. hexapetalus* genome. An unrooted maximum likelihood (ML) tree was then constructed based on the protein sequences of all *A. hexapetalus* P450s (AhP450s) and *A. thaliana* P450s (AtP450s). Phylogenetic analysis revealed that AhP450 genes were divided into nine clans (CYP51, 71, 72, 74, 85, 86, 97, 710 and 711), including 42 families (Fig. 7a; Table S21). In order to provide insight into the biosynthesis of *A*. *hexapetalus* pharmacologically active oxidized sesquiterpenes such as YZSA, phylogenetic analysis was constructed between AhP450s and 23 P450s involved in plant sesquiterpenes biosynthesis (Fig. 7b). Most of the P450s involved in modifying plant sesquiterpenes belong to the CYP71 family [37]. However, there are also a few enzymes associated with sesquiterpene oxidation that have been identified in other families, such as CYP76 [54–56], CYP82 [57], CYP701 [56], CYP706 [58, 59], and CYP736 [60]. Notably, all of these families are classified within the CYP71 clan. Therefore, AhP450s in these families, especially in the CYP71 family, deserve particular attention. Additionally, combined with the results of gene family expansion analysis mentioned above that *A*. *hexapetalus* CYP76 family expanded after divergence from *A. cherimola* (Table S17), CYP76 family has also generated further research interest.

Recently, two 2-ODDs, FtmOx1 from *Aspergillus fumigatus* [41] and NvfI from *Aspergillus novofumigatus* [42], were reported to form endoperoxide, which is an important step in the formation of YZSA. The 2-ODD families of *A. hexapetalus* were analyzed, and 92 2-ODD genes were identified from the genome using the reported HMM model (PF03171) [61]. 2-ODD gene family is the second largest enzyme family in the plant genome divided into three subfamilies according to amino acid sequence similarity: DOXA subfamily for primary metabolism, DOXB subfamily for post-translational modifications of peptide chains and DOXC subfamily for plant secondary metabolism including phenolic acids, alkaloids and terpenoids [38, 62]. Here 81 DOXC genes containing both classic 2OG-FeII Oxy (PF03171) domain and conserved DIOX N (PF14226) domain [63] were further identified as candidate enzymes that may be involved in YZSA generation (Table S22).

### Discovery of terpenoid biosynthetic gene clusters

**Fig. 8:**
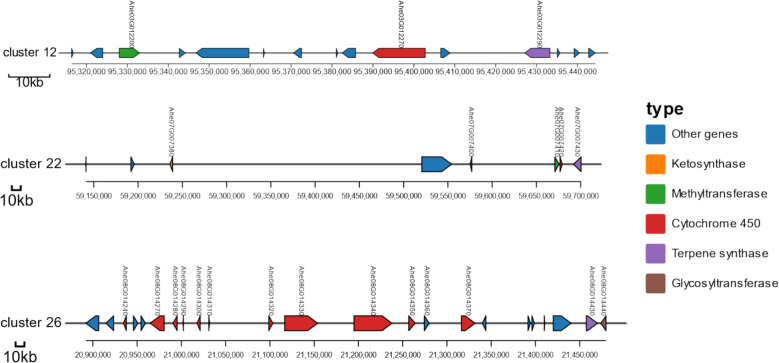
Discovery of terpene biosynthetic gene clusters in *A*. *hexapetalus* genome. The sesquiterpene, monoterpene and triterpene putative terpene biosynthetic gene clusters found in the genome of *A*. *hexapetalus*

In recent years, it has been reported that TPSs and post-modifying enzymes may cluster together in plant genomes and catalyze continuous biosynthetic steps in terpenoid biosynthesis [64, 65]. To determine whether *A*. *hexapetalus* contains potential terpenoid biosynthetic gene clusters (BGCs), the chromosome-scale genome of *A*. *hexapetalus* was analyzed on plantiSMASH. A total of 26 secondary metabolite clusters have been identified, including 8 terpenoid-related biosynthetic gene clusters (Table S23), among them three gene clusters simultaneously contain TPSs and oxygenases P450s (Fig. 8). A 129 kb genomic region on chromosome 3 contained a sesquiterpene synthase and a putative member of CYP88 family. In addition, a 692.83 kb putative monoterpene related cluster was detected on chromosome 7, including a monoterpene synthase clustered together with two genes encoding enzymes of CYP71 family. 13 genes were also organized as a cluster in a 610.53 kb stretch on chromosome 8. This cluster contained a triterpene synthase flanked by 11 P450 paralogues of CYP81 family, which were divided into 3 groups with the identity in each group at least 82.99%, suggesting a P450 tandem duplication event.

## Discussion

With the increased availability of genomic tools, essential and vital basic resources are provided for the study of plant gene function, population genetics, evolution, and breeding. Since the publication of the first plant reference genome of *Arabidopsis thaliana* in 2000 [66], benefiting from notable improvements in sequencing technology and computational algorithms for genome assembly, thousands of plant genomes have been sequenced and assembled [67]. In recent years, most of the assembled genomes used third-generation sequencing (TGS) technology, characterized by its capability of generating very long reads to overcome the drawback of short reads of next-generation sequencing (NGS). In this study, we performed PacBio third-generation sequencing and assembly on *A. hexapetalus*, resulting in a genome sequence of 915.15 Mb. This genome size was similar to the mean value of DNA C-values of other species in the Annonaceae (1.08 pg) [68]. Eventually, the genome sequences with 911.58 Mb in length were assembled onto the eight expected chromosomes and the BUSCO result indicated that the genome assembly generated in this work is a nearly complete high-quality genome.

The high-quality genomic resources generated here will facilitate an understanding of phylogenetic position and diversity of *A. hexapetalus* and other members of Annonaceae family. Here, phylogenetic analysis suggested the divergence between the genera *Artabotrys* and *Annona* happened at approximately 45.77 Mya and the Annonaceae diverged from other Magnolia species about 103.58 Mya. An ancient WGD event discovered in the *A. hexapetalus* genome, is one of the few reported for the Magnoliids as a whole, presenting several intriguing results for discussion. In previous study, the WGD events observed in both Cinnamomum and Liriodendron subsequent to their most recent common ancestor (MRCA) with Annona [69], however, this study found that WGD events in *A. hexapetalus* is earlier than the MRCA of Annona and Magnoliaceae (Figs. 3c and d). The other five Magnoliaceae species share a common WGD event with *A. hexapetalus* around 1.3 Ks units. Due to a recent WGD event in these Magnoliaceae species, the loss of genes from the earlier duplication event has accelerated, making the traces of the earlier duplication events increasingly less distinct and the peaks tend to appear relatively flattened. New evidence could help deepen the understanding of evolutionary relationships in the Magnoliales. Meanwhile, in this research the analysis result shows the WGD events in *Annona cherimola* with a major peak (Ks = 0.12, 4dtv = 0.01) and a minor peak (Ks = 1.43, 4dtv = 0.49, shared with *A. hexapetalus*) (Figs. 3a and b), inconsistent with previous study that noted the absence of a WGD event in *Annona cherimola* [24], warranting further investigation. At the same time, the limitations of Ks plot analyses should also be acknowledged: Ks plots can be difficult to interpret, and the accuracy of WGD identification from Ks plots seems contentious.

Not only do the roots of *A. hexapetalus* possess antimalarial medicinal properties, but its flowers also contain ethereal oils ranging from 0.75% to 1%, which can be used as raw materials for perfumes and soaps, thus *A. hexapetalus*-derived terpenoids hold considerable value. Actually, genome research provides insight into active natural products biosynthesis. Recently, a chromosome-level genome of *Taxus chinensis* var. *mairei* has been reported and facilitated the elucidation of the biosynthesis of paclitaxel, an anti-cancer hot spot molecule [70]. According to *A. hexapetalus* chromosome-level genome assembled in this work, significant expansion of gene families involved in biosynthesis of secondary metabolites was found, especially the TPS and cytochrome CYP76 families, which likely play a significant role in the production of specific terpenes in *A. hexapetalus*. Furthermore, a total of 57 TPSs were identified in this study, most of them were from either TPS-a or TPS-b families, implying that sesquiterpene and monoterpene metabolites in *A. hexapetalus* are highly diverse. A remarkable feature of TPS gene arrangement on chromosomes is its organisation as tandem clusters, which is the consequence of local gene tandem duplication.

The challenge of elucidating the biosynthetic pathway of complex terpenoids pertains to the identification of candidates involved in the downstream pathway of terpene. Genome sequencing enabled the identification of 310 members from the CYP superfamily, which are divided into 42 families in nine clans. Phylogenetic analysis was further constructed between AhP450s and 23 P450s involved in plant sesquiterpene biosynthesis and revealed the range of enzymes that may be involved in this process. Besides, 2-ODDs aroused research interest as two 2-ODDs have been reported to form endoperoxide [41, 42]. 92 2-ODD genes were identified and 81 of them belong to DOXC subfamily for plant secondary metabolism.

It is common that homologous genes with a high sequence identity cluster in plant genomes [71]; in recent years, some functionally related non-homologous genes were also found to form gene clusters. Unlike the BGCs found in bacteria and fungi, BGCs discovered in plants often contain only part of the biosynthetic pathway of metabolites [72]. Three putative terpene biosynthetic gene clusters that catalyze the generation of monoterpene, sesquiterpene, and triterpene respectively were also discovered in the genome of *A. hexapetalus*. The BGCs discovered in our study confirm that TPS/P450 gene pairs can be clustered together in plant genomes, thus deepening the current understanding of metabolite diversity of *A. hexapetalus*.

## Conclusion

In summary, a high-quality chromosome-level genome assembly for *A. hexapetalus* was successfully generated, providing a robust genomic framework that enhances understanding of its evolutionary dynamics. This reference genome serves as a crucial resource for identifying potential genes involved in the biosynthesis of pharmacologically active terpenoids. Taken together, this work lays the groundwork for future research aimed at sustainable utilization of this valuable species.

## Methods

### Plant material and DNA extraction

For genome sequencing, we collected dozens of *Artabotrys hexapetalus* plants from the wild in Changjiang Li Autonomous County, Hainan Province. No specific licenses or permissions from the local government were required for our collection. The voucher specimen (ID-S-3007) was identified by Prof. Lin Ma (Department of Natural Products Chemistry, Institute of Materia Medica, Chinese Academy of Medical Sciences) and deposited in the herbarium at institute of Materia Medica, Chinese Academy of Medical Sciences (CAMS). Fresh and healthy individuals of *A. hexapetalus* were harvested and immediately frozen in liquid nitrogen, followed by preservation at − 80^◦^C in the laboratory prior to DNA extraction. High-quality genomic DNA was extracted from young leaves using a modified CTAB method [73]. RNase A was used to remove RNA contaminants.

### Estimation of genome features

The short-reads from Illumina platform were quality filtered by fastp [74] using the parameters -q 10 -u 50 -y -g -Y 10 -e 20 -l 100 -b 150 -B 150. The quality filtered reads were used for genome size estimation. We counted the 85,690,154,293 kmers with Jellyfish [75] software and calculated the genome characteristics using Genomescope [76] software.

### Genome sequencing

For Illumina sequencing, a short-read (350 bp) library was constructed and sequenced on Illumina Novaseq platform (Illumina, CA, USA), and 102.26 Gb of clean reads were obtained. For PacBio sequencing, genomic DNA was fragmented to construct a long-read library according to the manufacturer’s instructions (Pacific Biosciences, CA, USA), and then the library was sequenced on PacBio Sequel II platform. After filtering out the low-quality reads and sequence adapters, we obtained 72.62 Gb of clean subreads with an N50 value of 18.39 Kb.

Short reads generated from the Illumina platform were used for the estimation of the genome size, the level of heterozygosity and repeat content of the genome, and long reads from the PacBio platform were used for genome assembly.

### PacBio CCS library construction

Genomic DNA was sheared into approximately 15 kb fragments by Megaruptor. The SMRTbell library was constructed using the SMRTbell Express Template Prep kit 2.0 (Pacific Biosciences). Briefly, 10 µg of the sheared DNA was carried into the first enzymatic reaction to remove single-stranded overhangs followed by treatment with repair enzymes to repair any damage that may be present in the DNA backbone. After DNA damage repair, ends of the double-stranded fragments were polished and subsequently tailed with an A-overhang at the 3’end. Ligation with T-overhang SMRTbell adapters was performed at 20 °C for 60 min. Following ligation, the SMRTbell library was digested by exonuclease and purified with 0.45X AMPure PB beads. The size distribution and concentration of the library were assessed using the FEMTO Pulse automated pulsed-field capillary electrophoresis instrument (Agilent Technologies, Wilmington, DE) and the Qubit 3.0 Fluorometer (Life Technologies, Carlsbad, CA, USA). Following library characterization, 3 µg was subjected to a size selection step using the Sage ELF system (Sage Science, Beverly, MA) to collect SMRTbells 15 18kb. After size selection, the library was purified with 1X AMPure PB beads. Library size and quantity were assessed using the FEMTO Pulse and the Qubit dsDNA HS reagents Assay kit. Sequencing primer and Sequel II DNA Polymerase were annealed and bound, respectively, to the final SMRTbell library. The library was loaded at an on-plate concentration of 55 pM using diffusion loading. SMRT sequencing was performed using a single 8 M SMRT Cell on the Sequel II System with Sequel II Sequencing Kit, 1800-min movies by Pacific Bioscience (USA).

### Genome assembly and annotation

PacBio clean data was corrected with the help of Canu [77] software, and then assembled using Smartdenovo [78] software based on the corrected data. Illumina data was used to perform three rounds of correction by Pilon [79] software, and finally we obtained 915.15Mb genome sequence with a Contig N50 value of 3.94Mb.

We integrated three approaches, namely, de novo prediction, homology search, and transcript-based assembly, to annotate protein-coding genes in the genome. The de novo gene models were predicted using two ab initio gene-prediction software tools, Augustus (v3.1.0) [80] and SNAP (2006–07–28) [81]. For the homolog-based approach, GeMoMa (v1.7) [82] software was used with reference gene models from the other species. For the transcript-based prediction, RNA-sequencing data was mapped to the reference genome using Hisat (v2.1.0) [83] and assembled by Stringtie (v 2.1.4) [84]. GeneMarkS-T (v5.1) [85] were used to predict genes based on the assembled transcripts. The PASA (v2.4.1) [86] software was used to predict genes based on the unigenes (and full-length transcripts from the PacBio (ONT) sequencing) assembled by Trinity (v2.11) [87]. Gene models from these different approaches were combined using the EVM software (v1.1.1) [88] and updated by PASA. In total, 23,859 protein-coding genes with an average length of 5626.98 bp were predicted in the assembled *A. hexapetalus* genome.

### Anchoring contigs in the *A. hexapetalus* genome using Hi-C technology

We collected *A*. *hexapetalus* branches with apical buds ready to emerge, surrounded by several young leaves, for the Hi-C project. To anchor contigs, 443,616,577 clean reads pairs were generated from the Hi-C library and were mapped to the polished *A. hexapetalus* genome using BWA (bwa-0.7.17) [89] with the default parameters. Paired reads with mate mapped to a different contig were used to do the Hi-C associated scaffolding. Self-ligation, non-ligation and other invalid reads, such as Start NearRsite, PCR amplification, random break, Large Smal Fragments and Extreme Fragments, were filtered. Lachesis [90] was further applied to order and orient the clustered contigs. Finally, 911.58 Mb of the assembled genome were anchored into 8 chromosomes.

### Functional annotation of protein-coding genes

Gene functions were inferred according to the best match of the alignments to the National Center for Biotechnology Information (NCBI) Non-Redundant (NR), EggNOG [91], KOG, TrEMBL [92], GO and Swiss-Prot [92] protein databases using diamond blastp (diamond v0.9.29.130) and the Kyoto Encyclopedia of Genes and Genomes (KEGG) database [93] with an E-value threshold of 1E-3. The protein domains were annotated using InterProScan (v5.34–73.0) [94] based on InterPro protein databases. The motifs and domains within gene models were identified by PFAM databases [95]. Gene Ontology (GO) IDs for each gene were obtained from TrEMBL, InterPro and EggNOG. In total, approximately 97.06% of the predicted protein-coding genes of *A*. *hexapetalus* could be functionally annotated with known genes, conserved domains, and Gene Ontology terms.

### Annotation of non-coding RNA genes

We used tRNAscan-SE (v1.3.1) [96] algorithms with default parameters to identify the genes associated with tRNA, which is an adaptor molecule composed of RNA used to bridge the three-letter genetic code in messenger RNA (mRNA) with the twenty-letter code of amino acids in proteins. For rRNA identification, we used barrnap (v0.9) (https://github.com/tseemann/barrnap) with default parameters to identify the genes associated with rRNA. SnoRNA is a class of small RNA molecules that guides chemical modifications of other RNAs, mainly ribosomal RNAs, transfer RNAs and small nuclear RNAs. MiRNAs and snRNAs were identified by Infernal (v1.1) [97] software against the Rfam (v14.5) [98] database with default parameters.

### Annotation of repetitive sequences

Transposon element (TE) and tandem repeat were annotated by the following workflows. TE were identified by a combination of homology-based and de novo approaches. We first customized a denovo repeat library of the genome using RepeatModeler [99] (http://www.repeatmasker.org/RepeatModeler/), which can automatically execute two de novo repeat finding programs, including RECON [100] and RepeatScout [101]. Then full-length long terminal repeat retrotransposons (fl-LTR-RTs) were identified using both LTRharvest (v1.5.9) [102] and LTR finder (v2.8) [103]. The high-quality intact fl-LTR-RTs and non-redundant LTR library were then produced by LTR retriever [104]. The flanking sequences on both sides of LTR were extracted, compared by MAFFT (v7.205) [105], and the distance was calculated by Kimura model in EMBOSS (v6.6.0) [106]. The formula for calculating the time is T = K/(2 × r) with the molecular clock r 7 × 10^9^ [107].

Non-redundant species-specific TE library was constructed by combining the de novo TE sequences library above with the known Dfam (v3.5) database. Final TE sequences in *A*. *hexapetalus* genome were identified and classified by homology search against the library using RepeatMasker (v4.12) [108]. Tandem repeats were annotated by Tandem Repeats Finder (TRF 409) [109] and MIcroSAtellite identification tool (MISA v2.1) [110].

### Gene family identification

Protein-coding sequences from the genomes of *A*. *hexapetalus* and other 8 closely related species were compared using OrthoFinder (v2.4.0) software [111] to cluster their families. The Pfam V33.1 [95] database was used to annotate the obtained gene families. As a result, 33197 gene families were identified in this work and 2272 genes were unique to *A*. *hexapetalus*.

### Phylogenetic analysis

To reveal phylogenetic relationships among *A*. *hexapetalus* and other closely related species, single-copy ortholog genes were used for phylogenetic tree construction. The protein sequences of the single-copy ortholog genes were aligned with MAFFT (v7.205) [105] program, and the corresponding Coding DNA Sequences (CDS) alignments were generated and concatenated with the guidance of protein alignment. ModelFinder [112], a model detection tool built with IQ-TREE, was used to detect the model, and the best model was obtained as JTT + F + G4, and then the ML method was used to construct the evolutionary tree with 1000 bootstrap replicates. Then, the divergence time was calculated using MCMCTREE, the software package that came with PAML v4.9i software [113].

### Gene family expansion and contraction analysis

Based on the identified gene families and the constructed phylogenetic tree with predicted divergence time of those species, we used CAFE (v4.2) [112] to analyze gene family expansion and contraction. In CAFE, a random birth and death model is proposed to study gene gain or loss in gene families across a specified phylogenetic tree. Then, conditional *p*-value was calculated for each gene family, and family with conditional *p*-value less than 0.05 was considered to have an accelerated rate for gene gain or loss. These expanded and contracted gene families in *A*. *hexapetalus* (*p*-value ≤ 0*.*05) were mapped to KEGG pathways for functional enrichment analysis, which was conducted using the enrichment methods. This method implemented hypergeometric test algorithms and the Q-value (FDR, False Discovery Rate) was calculated to adjust the *p*-value using R package (https://github.com/StoreyLab/qvalue).

### Whole genome duplication analysis

WGD, also known as polyploidy, is the process of doubling the entire genome. At present, the Ks (synonymous mutation rate) method and 4DTv (fourfold synonymous third-codon transversion rate) method are commonly used to identify WGDs. Syntenic blocks and syntenic genes were identified using JCVI. Here, the synonymous substitution rate (*Ks*) values were calculated using wgd (v3.7.3) [114] software and the distribution of the *Ks* values was plotted. And the 4DTv values for all the orthologous and paralogous gene pairs were calculated and corrected using a custom script (https://github.com/JinfengChen/Scripts) to identify WGD events in *A. hexapetalus*.

### Analysis of TPS and oxygenase gene in* A. hexapetalus* genome

Terpene synthase and oxygenase genes were identified using the Hidden Markov Model in the PFAM database [115]. Subsequently, multiple sequence alignments of these proteins were performed using MEGA7 software [116]. The TPS phylogenetic tree was constructed using the Neighbor-Joining method [117] by MEGA7, and P450 phylogenetic tree was constructed using the ML method by FastTree [118]. The bootstrap process was replicated 1000 times [119]. Finally, the management and annotation of phylogenetic trees were accomplished using Itol (https://itol.embl.de/) [120]. The protein sequences of *A*. *thaliana* P450s were downloaded from Arabidopsis Cytochromes P450 (http://www.p450.kvl.dk/p450.shtml).

### Genome mining for terpenoid biosynthetic gene clusters

The plantiSMASH web server (default parameters) [121] was used to discover putative biosynthetic gene clusters (BGCs) in the *A*. *hexapetalus* genome. The FASTA sequences of the assembled genome along with their GFF3 (Generic Feature Format version 3) annotation files were supplied to the web server. Three putative terpenoid biosynthetic gene clusters were discovered through analysis of FASTA + GFF3 format input files. Finally, the management and annotation of terpenoid biosynthetic gene clusters were accomplished using ChiPlot (https://www.chiplot.online/).

## Supplementary Information

Additional file 1: Figure S1. Estimate of genome size of *A*. *hexapetalus *K-mer Depth Genome size (Mb). Figure S2. Hi-C heat map of *A*. *hexapetalus *chromosome interactions. Figure S3. Distribution maps of genes derived from the three prediction methods after integration. Figure S4. LTR insertion events in *A*. *hexapetalus*. Figure S5. Phylogenetic tree with percentage supported by bootstrap detection. Figure S6. The GO functional enrichment of *A*.*hexapetalus *unique genes. Figure S7. The dot plot of KEGG functional enrichment of *A*. *hexapetalus*unique genes. Figure S8. The dot plot of KEGG functional enrichment of *A*. *hexapetalus *unique genes. Figure S9. Distribution map of TPSs in *A*. *hexapetalus *chromosomes. Fig. S10.Conserved motif analysis of TPS subfamily in *A. hexapetalus*. Table S1. Genome size estimation based on different K-mer of *A*.*hexapetalus *genome. Table S2. Summary of the long reads sequencing of *A*. *hexapetalus *genome. Table S3. Statistics of *A*. *hexapetalus *second-generation data comparison. Table S4. Quantification of the mapped Hi-C produced clean reads of *A*. *hexapetalus *genome. Table S5. Genome information of *A*. *hexapetalus *after Hi-C assembly. Table S6. Genome intergrity estimation of *A*.*hexapetalus *genome. Table S7. Statistics of *A*. *hexapetalus *genome coding gene prediction results. Table S8. Comparison of *A*. *hexapetalus *gene set with other species. Table S9. Database and the corresponding annotated genome of *A*. *hexapetalus*. Table S10. Statistics of *A*. *hexapetalus*genome Noncoding RNAs. Table S11. Statistics of *A*. *hexapetalus *genome transposable elements. Table S12. Statistics of *A*. *hexapetalus *genome tandem repeats. Table S13. Angiosperm species in this study. Table S14. Statistics of *A*. *hexapetalus *genome syntenic gene pairs. Table S15. Statistics of *A*. *hexapetalus*gene family cluster. Table S16. The GO enrichment analysis of expanded genes in *A*. *hexapetalus*. Table S17. Statistics of *A*. *hexapetalus *expansion and contraction of gene families. Table S18. Reported TPSs belonged to different TPS subfamilies used to identify the members of the TPS gene subfamily. Table S19. Statistics of *A*.*hexapetalus *TPS subfamilies distribution. Table S20. Tandem duplicated TPS gene clusters. Table S21. Statistics of *A*. *hexapetalus *P450 family distribution. Table S22. List of *A*. *hexapetalus*2-OGGs in DOXC subfamily. Table S23. Statistics of *A*. *hexapetalus *secondary metabolite biosynthetic gene clusters.

## Data Availability

The data that support the findings of this study have been deposited into CNGB Sequence Archive (CNSA) of China National GeneBank DataBase (CNGBdb) with project accession ID: CNP0006579.
