# Supplementary material for: Chromosome-scale genome assembly of Artabotrys hexapetalus provides insights into its evolution and terpenoid biosynthesis
Source: BMC Plant Biol. 2026 Aug 12;26:1377. doi: 10.1186/s12870-025-07645-w (PMC13471500; doi:10.1186/s12870-025-07645-w)
Supplement: Supplementary file 1 [file 12870_2025_7645_MOESM1_ESM.zip › Additional file/Additional file 1new.docx]

**Fig. S1** Estimate of genome size of *A*. *hexapetalus K-mer* Depth Genome size (Mb).

**Fig. S2** Hi-C heat map of *A*. *hexapetalus* chromosome interactions. The heat map presents the Hi-C chromosomal interactions (300 kb resolution). The x- and y-axes present the order of the positions of scaffolds on the corresponding pseudochromosomes.

**Fig. S3** Distribution maps of genes derived from the three prediction methods after integration.

**Fig. S4** LTR insertion events in *A*. *hexapetalus*

**Fig. S5** Phylogenetic tree with percentage supported by bootstrap detection.

**Fig. S6** The GO functional enrichment of *A*. *hexapetalus* unique genes.

**Fig. S7** The dot plot of KEGG functional enrichment of *A*. *hexapetalus* unique genes.

**Fig. S8** The dot plot of KEGG functional enrichment of *A*. *hexapetalus* unique genes.

**Fig. S9** Distribution map of TPSs in *A*. *hexapetalus* chromosomes.

**Fig. S10** Conserved motif analysis of TPS subfamily in *A. hexapetalus*. (a) DDXXD motif in TPS-a subfamily. Four TPS-a subfamily members lack C-terminal regions and consequently lack the DDXXD motif. (b) DDXXD motif in TPS-b subfamily. (c) RRX8W motif in TPS-b subfamily. Six TPS-b family members lack this relatively conserved motif. (d) DXDD motif in TPS-c subfamily. (e) DDXXD motif in TPS-e/f subfamily. (f) DDXXD motif in TPS-g subfamily.
